# Transparency, trust, and community welfare: towards a precision public health ethics framework for the genomics era

**DOI:** 10.1186/s13073-020-00800-y

**Published:** 2020-11-20

**Authors:** Eric T. Juengst, Annelies Van Rie

**Affiliations:** 1 Center for Bioethics, School of Medicine, University of North Carolina at Chapel Hill, 333 MacNider Hall, Chapel Hill, NC 27599-7240 USA; 2 Family Medicine and Population Health, Faculty of Medicine, University of Antwerp, Campus Drie Eiken R232, Universiteitsplein 1, 2610 Wilrijk, Belgium

**Keywords:** Pathogen sequencing, Genomic epidemiology, Precision public health, Ethics

## Abstract

Infectious disease control is experiencing a paradigm shift, as pathogen sequencing technologies and digital applications are increasingly implemented for control of diseases such as tuberculosis, Ebola, and COVID-19. A new ethical framework should be a critical part of this emerging paradigm to ensure that the benefit of precision public health interventions based on advances in genomics research is not outweighed by the risks they pose to individuals, families, and vulnerable segments of the population. We suggest that the ethical framework guiding practice in this domain combines standard precepts from public health ethics with emerging ethics principles from precision medicine.

## Precision public health

Precision public health was first defined by the Health Department of Western Australia as “the application and combination of new and existing technologies to more precisely describe and analyse individuals and their environment, tailor preventive interventions for at-risk groups, and improve the overall health of the population” [1]. Using sequencing data of pathogens can result in unprecedented levels of speed and accuracy of contact and source investigations. Digitalizing and sharing this data across public health programs can expedite anticipatory planning and interventions [2]. These developments pose new ethical challenges, since collecting and using genomics data on pathogens to target populations for public health interventions requires negotiations between individual rights, target group interests, and the larger public welfare. While none of the relevant concerns—personal privacy risks, risks of group harms through stigmatization and blame, or population health—are foreign to public health, the efficiency and power of precision public health methods can both fuel and focus them in unprecedented ways. Here we discuss how traditional public health ethics and the ethos of genomic medicine can come together to form a hybrid ethical framework for precision public health, adapted to the big data challenges of the twenty-first century.

## Public health ethical principles

Four traditional principles from public health ethics help lay the foundations for the use of precision tools like pathogen sequencing. The *priority principle* ensures that health issues that threaten social stability supersede individual interests, justifying interventions such as quarantining. The *harm principle* allows public health authorities to restrict personal liberties for disease control without the consent of individuals or groups involved, as is currently often the case for disease surveillance or contact tracing. The *least intrusive means* and *social justice* principles serve as the traditional brakes on public health's utilitarian goals. Using the *least intrusive means* necessary to mitigate the impact of any autonomy violations from necessary public health interventions. The *social justice principle* requires the burdens and benefits of public health interventions to be distributed fairly across all sectors of society, even at the expense of disease control goals [3].

In democratic societies, the translation of the traditional principles of public health ethics into public health law and policy is based on a population’s decision to voluntarily trade certain liberties for protection against health threats. For marginalized groups without a voice in that public decision-making, this trade-off may be perceived as an oppressive imposition of social risk. As phylogenetic surveillance for HIV, TB, and COVID-19 is starting to show, this problem is amplified as precision approaches to public health data-gathering make community targeting more precise and powerful [4]. This is where the ethics of precision medicine can help.

## Precision medicine ethical principles

The ethics of precision medicine combines the commitments of traditional medical ethics to patient privacy and autonomy with the concerns for group health interests and disparities raised by genomic risk stratification [5]. The ethos of precision medicine focusses on the importance of *personal and community control* over the generation and disposition of identifiable genomic information, the need to consider the *rights and interests of other people affected by a patient’s genomic information*, the obligation to avoid harm by protecting the *privacy of identifiable stored genomic information* and the importance of *professional transparency* about information revealed by genomic screening, including disclosure of clinically actionable findings [6].

## A hybrid framework

Precision public health ethics lies at the intersection between the clinical patient-doctor covenant of genomic precision medicine and the public decision-making that empowers action by public health authorities, thereby creating a hybrid ethical framework. In this intersection, the application of the traditional principles of public health ethics is influenced by the need to be responsive to the personalizing power of big data. While the goal of precision public health interventions remains at the level of population disease control, the focus of the ethical concern shifts from the population as a whole to at risk communities, individuals, and locations. The resulting hybrid principle of c*ommunity priority* entails that the risks posed by any targeted precision public health intervention should be justified by the importance of the disease burden in the targeted group [7].

The engagement of both individuals and groups in precision medicine decision-making regarding identifiable and socially potent information also affects the ways in which the Harm Principle is deployed. As precision public health targets particular locations, groups, or individuals, the need to enlist the people at risk as partners in intervention planning and implementation increases. The hybrid principle of *shared authority* is especially important when those targeted lack a meaningful voice in the democratic ratification of public health authority. *Shared authority* reflects the recognition that a socially just public health system should complement the public’s endorsement of authorizing legislation with transparent public engagement when particular groups are targeted for intervention [8].

Another important contribution of precision medical ethics to precision public health ethics is its robust commitment to information privacy, good governance of data, and professional confidentiality. In public health, the exchange of surveillance data is critical to combatting the spread of disease. The social risks of more precise information, such as whole genome sequences of pathogens linked to clinical and sociodemographic meta-data (such as concurrent infections, geographic location, immigration status, or history of incarceration) requires expanding the public health principle of *least intrusive means* from the domain of disease control interventions to the realm of data collection and data sharing. The hybrid *least intrusive data use* principle dictates that, to prevent the public disclosure of potentially stigmatizing information, all data sharing should occur within confidential contexts protected by strong data security practices and conform to relevant personal privacy laws [9].

Finally, the ethics of precision medicine embraces the commitment of public health ethics to social justice and places an emphasis on professional transparency and the trust it engenders. Transparent communication of precision public health information that is free of moralizing language will be needed to prevent inaccurate public attributions of responsibility for disease hotspots and stigmatization of people, locations, communities, or groups that are already socially marginalized. Precision public health communication will also have to be explicit about its scientific limitations and emphasize the permeability of targeted locations or communities. Just as clinical transparency about actionable genetic risks can empower patients to become involved in preventive care, *proactive transparency* about precision public health efforts can help communities to become active partners in disease control efforts [10].

## Conclusions and outlook

In conclusion, most laws and guidelines that govern public health infectious disease control efforts were developed under historical conditions that greatly differ from our current societal context. The speed, precision, and efficiency of big data, digital technologies, and pathogen sequencing offer public health opportunities but come with the responsibility to adapt its practices to a world increasingly committed to privacy, respect for human rights in health matters, and social justice. One way forward for precision public health ethics is through a framework that integrates the key moral commitments and ethical requirements of precision medicine and traditional public health (Table 1). The recommendations based on this hybrid approach can help generate awareness of the ethical issues at stake and ensure that the implementation of precision public health actions is in line with bioethical principles, protect public health, promote solidarity, equity, and social justice, and empower and engage patients and their communities.

**Table 1:** The four intersectional elements of precision public health ethics

Hybrid principle	Key variable	Rationale	Relationship to precision medicine ethics	Relationship to public health ethics
Community health priority	Importance of public health problem	Public health as a prerequisite for individual heath	Unique interests and needs of targeted groups	Public priority principle
Shared authority	Severity of social risks to individuals and groups and value of local knowledge	Too much state control of individual and community choices undermines public trust	Personal and group control of identifiable information	Harm principle and the limits of the Ulysses contract
Least intrusive data use	Social potency of information	Informational security promotes trust and reduces harms	Respect for informational privacy and confidentiality	Least intrusive means principle
Proactive transparency	Levels of community solidarity and vulnerability	Transparency promotes trust and empowers community involvement	Professional transparency about actionable information	Social justice principle

## Data Availability

Not applicable
